# Magnesium degradation-induced variable fixation plates promote bone healing in rabbits

**DOI:** 10.1186/s10195-024-00803-0

**Published:** 2024-11-21

**Authors:** Jian Wen, Yu Zeng, Shenghui Su, Meiling Song, Zhe Wang, Xiaofan Chen, Xieping Dong

**Affiliations:** 1 JXHC Key Laboratory of Digital Orthopedics, Jiangxi Provincial People’s Hospital, The First Affiliated Hospital of Nanchang Medical College, 152 Aiguo Road, Nanchang, 330006 Jiangxi China; 2 Department of Pain Management, The 2Nd Affiliated Hospital, Jiangxi Medical College, Nanchang University, No.1 Minde Road, Nanchang, 330006 Jiangxi China; 3 Jiangxi University of Chinese Medicine, No. 1688, Meiling Avenue, Nanchang, 330004 Jiangxi China; 4 Ruijin Traditional Chinese Medicine Hospital, Ruijin, 342500 Jiangxi China

**Keywords:** Plate, Magnesium, Variable fixation, Axial micromotion, Osteogenesis, Femur fracture

## Abstract

**Background:**

Both initial mechanical stability and subsequent axial interfragmentary micromotion at fracture ends play crucial roles in fracture healing. However, the conversion timing of variable fixation and its effect on and mechanism of fracture healing remain inadequately explored.

**Methods:**

A magnesium degradation-induced variable fixation plate (MVFP) for femurs was designed, and its conversion timing was investigated both in vitro and in vivo. Then, locking plates and MVFPs with and without a magnesium shim were implanted in rabbit femur fracture models. X-ray photography and micro computed tomography (micro-CT) were performed to observe the healing of the fracture. Toluidine blue and Masson’s trichrome staining were performed to observe new bone formation. The torsion test was used to determine the strength of the bone after healing. Finally, reverse transcription-polymerase chain reaction (RT-PCR) and western blotting were used to detect the expression of osteogenesis-related genes in the three groups.

**Results:**

The MVFP with sample 3 magnesium shim showed greater axial displacement within 15 days in vitro, and its variable capability was likewise confirmed in vivo. X-ray photography and micro-CT indicated increased callus formation in the variable fixation group. Toluidine blue and Masson’s trichrome staining revealed less callus formation on the rigid fixation side of the locking plate, whereas the variable fixation group presented more callus formation, more symmetrical intraosseous calli, and greater maturity. The torsion test indicated greater torsional resistance of the healed bone in the variable fixation group. RT-PCR and western blotting revealed that the expression levels of BMP2 and OPG increased during early fracture stages but decreased in late fracture stages, whereas RANKL expression showed the opposite trend in the variable fixation group.

**Conclusions:**

MVFP promoted faster and stronger bone healing in rabbits, potentially by accelerating the expression of BMP2 and modulating the OPG/RANKL/RANK signaling axis. This study offers valuable insights for the clinical application of variable fixation technology in bone plates and contributes to the advancement of both internal fixation technology and theory.

*Level of evidence*: level V.

**Graphical Abstract:**

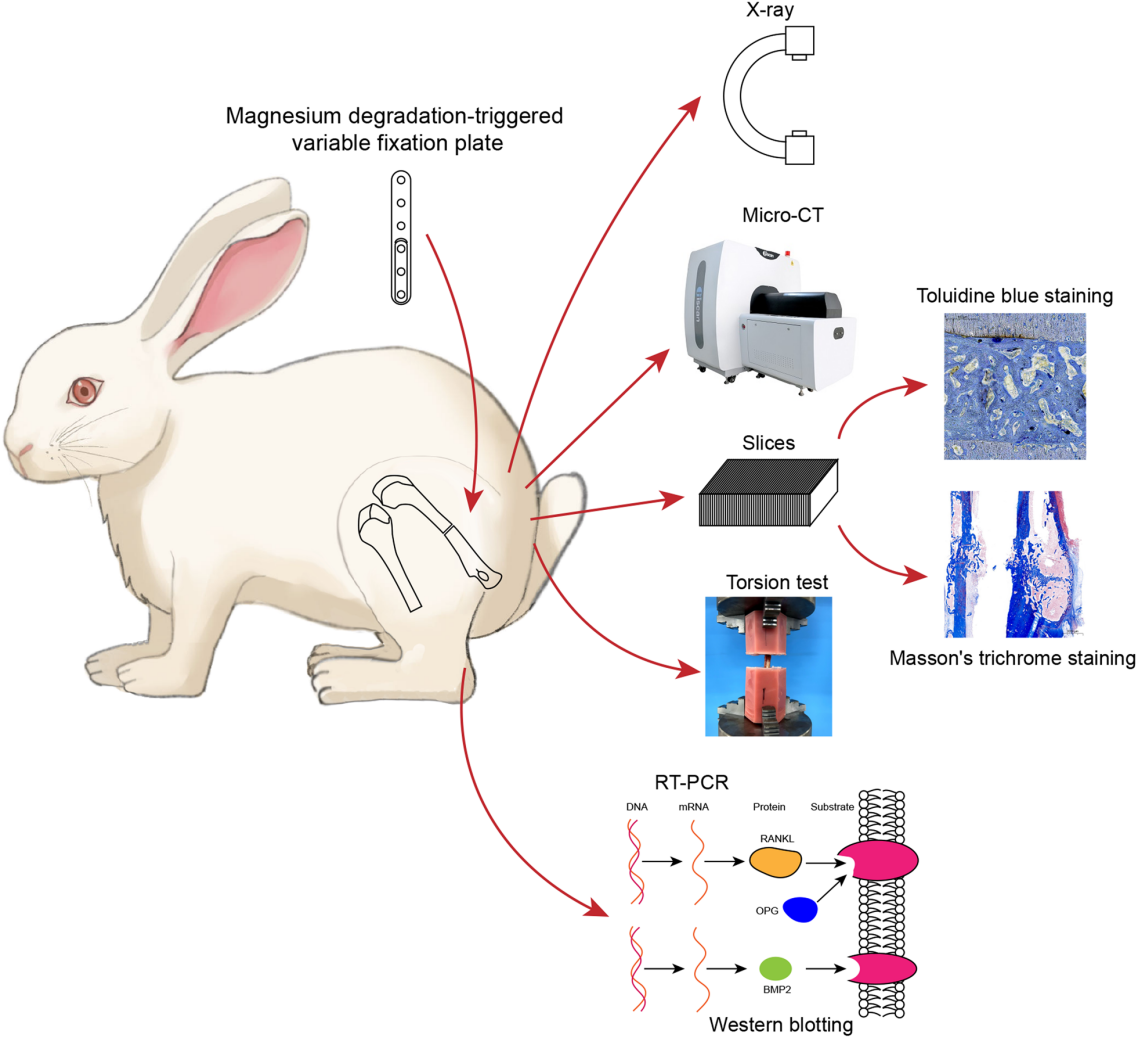

## Introduction

The global incidence of fractures is reported to be 11.6 per 1000 per year [1]. In 2019 alone, there were 178 million new fractures worldwide, and the cumulative number of fractures was 455 million [2]. Moreover, approximately 5–10% of patients with fracture are reported to experience nonunion or delayed union of the fracture [3]. The development of internal fixation techniques, including internal fixation instruments, can facilitate faster and more effective fracture healing [4, 5].

Since the initial utilization of plates for fracture fixation more than 100 years ago, significant advancements have been made; however, despite these improvements, many challenges still remain [6]. For example, the utilization of strong plates may lead to stress shielding, heightening the risk of delayed and nonunion fractures [7, 8]. This can further manifest as subplate osteoporosis and postremoval refracture. Moreover, asymmetric osteogenesis at the fracture end stabilized by locking plates (LPs) not only indicates that stress stimulation promotes callus growth but also reveals the limitations of traditional stress shielding associated with traditional plates [9].

It has been demonstrated that axial interfragmentary micromotion within a defined range of 0.2–1 mm is beneficial for fracture healing [9–12]. Han Z et al. suggested that the axial micromotion of a plate not only reduces stress shielding but also promotes more symmetrical fracture motion, thereby enhancing fracture healing [13]. To date, several novel plates that can achieve axial micromotion, such as dynamic locking screws, far cortical locking screws, active locking plates, biphasic plates, and axial micromotion locking plates, have been reported [13–18]. Many of these instruments have demonstrated favorable outcomes in preclinical studies, with some exhibiting satisfactory efficacy in clinical trials [17–19].

Moreover, multiple studies have shown that the initial mechanical stability of the fracture ends is crucial for the vascularization and differentiation of osteoblasts around the fracture ends [20–24]. Therefore, the variable fixation mode, which involves early stable fixation followed by an automatic transition to axial micromotion fixation, will theoretically better align with the mechanical stimulation needs at various stages of fracture healing. On the basis of this concept, we developed a novel magnesium degradation-induced variable fixation plate (MVFP) and evaluated its mechanical properties and effects on fracture healing in rabbits. This study offers an initial evaluation of the impact of MVFP on fracture healing, providing further validation for determining the optimal timing of axial micromotion of the interfragmentary. Additionally, the feasibility of creating distinct mechanical environments tailored to different stages of fracture healing using MVFP has been explored.

## Materials and methods

### Implant design

**Fig. 1:**
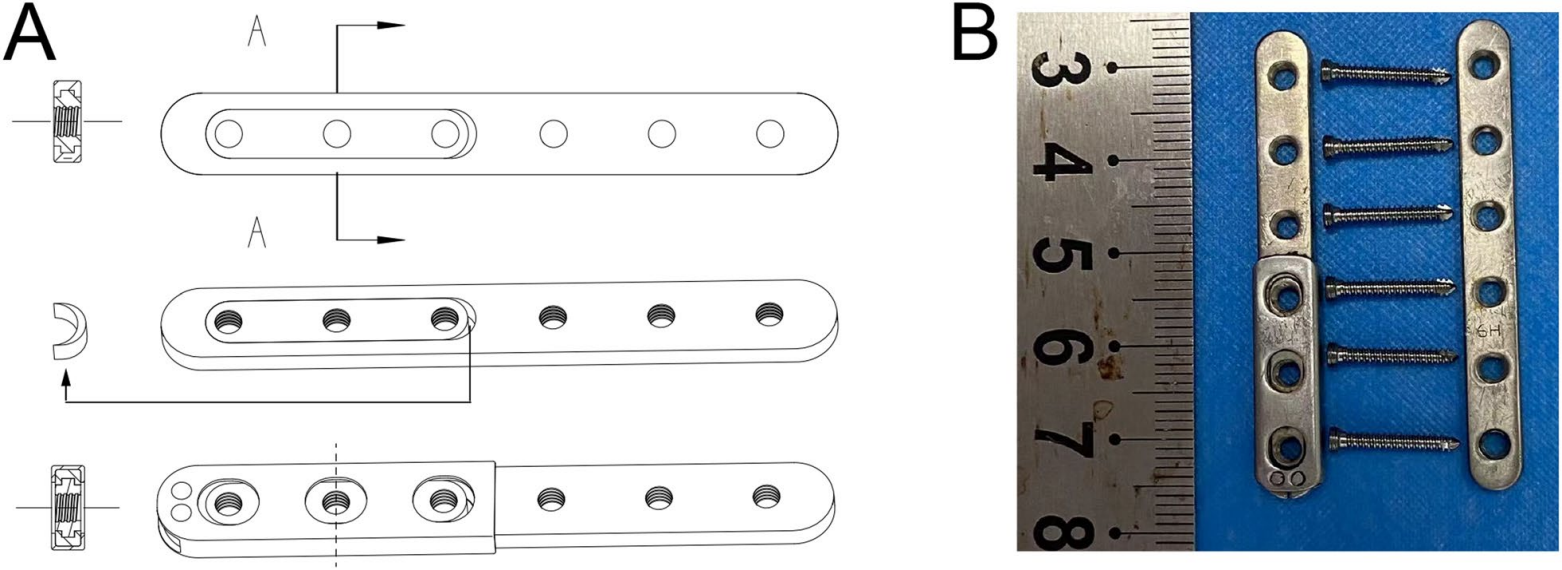
Appearance of a straight femoral LP and an MVFP in rabbits. **A** Design of the MVFP for rabbits. **B** Comparison of the appearances of a straight femoral LP and an equivalently specified MVFP for rabbits

A six-hole straight titanium alloy (Ti-6Al-4 V) femoral locking plate for rabbits, accompanied by 2 mm diameter locking screws, was designed [Suzhou Kangli Orthopaedics Instrument Co., Ltd., Suzhou, China; dimensions (length × width × thickness): 50 mm × 6 mm × 2 mm] (Fig. 1A). Additionally, for the MVFP, an additional slider featuring a lateral guide rail measuring 0.5 mm in depth and 1 mm in width [dimensions (length × width × thickness): 19.4 mm × 3.6 mm × 2 mm) was attached to one side of the plate. An axial 0.6 mm thick magnesium shim was inserted in the mid-gap between the slider and the plate to achieve variable fixation, leveraging its degradable properties. To prevent the slider from cutting out owing to the shallow depth of the lateral guide rail on the rabbit plate, we incorporated a 0.3 mm thick titanium envelope around the slider (Fig. 1B). This addition serves to restrict the slider’s movement in the vertical direction of the plate while ensuring unimpeded axial sliding.

To date, there is no universally established standard for the specific duration required for a stable mechanical environment in the early stage of fracture healing. Most literature suggests a range of 4–8 days for this period [20–24]. Therefore, a shim capable of achieving variable fixation of the MVFP within approximately 4–8 days after implantation was selected.

### Axial dynamization of the variable plate in vitro

In this study, three types of shims were designed, followed by an assessment of the average quality of these shims. After the shim was attached to the MVFP, it was secured onto a three-dimensional (3D)-printed polycarbonate stand, and a constant force of 3 kg was applied using a puller. The MVFP was subsequently immersed in a 250 ml beaker filled with phosphate buffered saline (PBS) solution in a 37 °C thermostatic water bath. The pH of the PBS solution was monitored every 12 h, and the solution was promptly replaced if it exceeded 7.45. The displacement of the slider was measured at 5, 10, and 15 days after immersion. Finally, the most suitable shim from the three types was selected on the basis of the displacement of the slider.

### Axial dynamization of the variable plate in vivo

The MVFP was implanted into a transverse femoral fracture rabbit model (right leg) with a 2 mm gap between the fracture ends (the detailed surgical procedures are described in the animal surgery section below). Lateral femoral radiographs of the affected limb were captured at 5 and 10 days postoperation to analyze the displacement of the MVFP slider. The animals were sacrificed 10 days after implantation, and the plates were removed to observe the axial dynamization of the slider and the condition of the shims.

### Grouping and animal surgery

In total, 72 adult male New Zealand White rabbits (Qingdao Kangda Biological Technology Co., Ltd., Qingdao, China), aged 6 months and weighing approximately 3 kg, were chosen for the study and randomly assigned to three groups of 24 rabbits each. Femoral fractures in the LP group were stabilized using an LP. In the axial micromotion locking plate (AMLP) group, femoral fractures were fixed with an MVFP without a magnesium shim. Femoral fractures in the MVFP group were fixed with an MVFP with a 0.6 mm magnesium shim.

Following a 1-week period of adaptive feeding, the rabbits were weighed and anesthetized via intramuscular injection of 4 mg/kg xylazine hydrochloride. The right thigh was shaved, and the rabbit was positioned on the animal operating table with the right side exposed.

Access to the lateral femur was achieved through the intermuscular space between the anterior and posterior groups of the thigh muscles. The periosteum was subsequently incised, and the lateral femoral muscles were subperiosteally peeled away using a periosteal stripper, exposing the lateral femur. The plate was then affixed to the lateral femur at the designated center point and secured with a bone holder. After four holes were drilled on the femur under the guidance of the locking sleeve, the plate was temporarily removed.

With the aid of a specialized osteotomy guide plate, a 2 mm segment of the middle femur was excised. The femur was then reset, the plate was reattached to the lateral side, and four self-tapping screws (Φ2 mm × 14 mm) were inserted through the original prefabricated holes. Following hemostasis, the surgical site was thoroughly rinsed and sutured.

### Radiographic evaluation

Overall, three animals were randomly chosen from each group, and X-ray examinations were conducted at 2, 4, 6, and 8 weeks postoperation to monitor the progress of fracture healing. The external callus area on the anteroposterior (AP) radiographs was identified and quantified using ImageJ software according to methods reported in literature [25, 26]. Given the strong correlation between bone mineral density and the mean gray value (MGV), we utilized the MGV to assess the bone mineral density (BMD) of the callus. The total gray value (TGV) was employed to quantify the amount of new bone, whereas the pixel count was used to evaluate the area of the callus projection on the AP radiographs. Owing to the less consistent photographic position and interference of the contralateral limb, quantitative calculation of the callus using lateral radiographs was deemed impractical.

### Micro computed tomography

Then, six animals from each group were subjected to micro computed tomography (micro-CT) examination, with three in each group being sacrificed at 4 weeks and the remaining three at 8 weeks. As the fracture had not fully healed at 4 weeks postoperation, removing the plate compromised the morphology of the sample. Consequently, the implants were retained during the micro-CT examination. However, owing to plate interference, calculating the new bone volume on the side proximal to the plate was not feasible. Therefore, the quantification of new bone volume was conducted solely on the contralateral half of the bone. By 8 weeks postoperation, the fracture had reached stability, prompting the removal of the plate during micro-CT to minimize its impact on the results. The volume, volume fraction and BMD of the region of interest (ROI) were recorded (ROI: a cylindrical region measuring 20 mm in length, centered at the fracture end and parallel to the long axis of the bone).

### Toluidine blue staining

After the completion of micro-CT scanning, the hard tissue sections of the samples were subjected to toluidine blue staining.

After fixation with 4% paraformaldehyde fixative for 48 h, the callus samples were dehydrated in a concentration gradient of ethanol, infiltrated with Technovit 7100 (KULZER, Munich, Germany, item no. 64709003) at 4 °C for 20 days, and polymerized with Technovit 7100 in a photocuring embedding machine (E520, EXAKT) for 12 h. Each callus sample was cut with a hard tissue microtome along the plane between the screws and the central line of the bone (E300CP, EXAKT, Norderstedt, Germany). The sections were polished with a hard tissue grinder (E400CS, EXAKT) to a thickness of 20–30 μm. Then, they were stained with toluidine blue for 5 min, washed with water, dehydrated in ethanol and xylene, and sealed with neutral gum.

### Bone demineralization and Masson’s trichrome staining

After fixation in 4% paraformaldehyde for 48 h, the samples were decalcified with 0.5 mol/L ethylenediaminetetraacetic acid solution at 25–30 °C with agitation at 110–120 revolutions per min. Following a 4-h rinse in running water, the samples were dehydrated in a gradient of alcohol and xylene and then embedded in paraffin. Tissue sections from the middle longitudinal section of the bone healing zone were obtained using a microtome (RM2016, Shanghai Leica Instrument Co., Ltd.) and mounted onto slides using a slide mounting machine (KD-P, Zhejiang Jinhua Cody Instrument Equipment Co., Ltd.).

After deparaffinization with xylene and a gradient of alcohol, the tissue sections were stained with Masson’s trichrome staining solution (Servicebio, Wuhan, China; catalog number: G1006-20ML), followed by rinsing and differentiation in 1% acetic acid. The samples were subsequently dehydrated with a gradient of alcohol and xylene, sealed with neutral gum, and examined under a microscope for photography.

### Torsion test

Torsion tests were performed to test the biomechanical properties of the new bones. Torsion stiffness, strength, and energy to failure were evaluated and compared after normalization to those of the contralateral healthy femur.

At 8 weeks after surgery, three animals from each experimental group were euthanized, and bilateral femoral specimens were collected. The samples were embedded with denture resin (Shanghai New Century Dental Department, Type II, Class I) at both extremities, with a 20 mm unembedded section along the femoral shaft. The original fracture defect area was located at the midpoint of the unembedded area. The position of the embedding on the contralateral healthy femur was referenced to the affected femur.

Torsion tests were conducted at room temperature with the samples moistened with normal saline. The testing parameters included a rotational speed of 10°/min until the sample fractured. The torsional strength corresponds to the maximum torque achieved during testing, whereas the torsional stiffness is determined by the linear slope of the torsion‒rotation curve until the maximum torsional torque is reached. The failure energy is calculated by integrating the area under the torsion‒rotation curve up to the peak torsional moment at which rupture occurred.

### Reverse transcription-polymerase chain reaction (RT-PCR)

RT-PCR was used to detect the expression levels of osteogenesis-related genes (*BMP2*, *OPG*, and *RANKL*) in the calli of the three groups. Bone callus was ground into powder with liquid nitrogen, and total RNA was extracted using a HyPure™ Bone Tissue RNA Extraction Kit (Beijing Genenode Biotech Co., Ltd., Beijing, China; catalog No: 2151A). The extracted RNA (1 μg) was reverse transcribed to cDNA using a Vazyme RNA Reverse Transcription Kit [HiScript^®^ II Q RT SuperMix for quantitative PCR (qPCR) (+ gDNA wiper), Vazyme, Beijing, China; catalog No: R223-01]. For RT-PCR, the reaction system was prepared according to the instructions provided with ChamQ SYBR qPCR Master Mix (Vazyme, catalog No: Q711-02) and amplified using a CFX Connect™ Real-Time PCR System (Bole Life Medical Products, Shanghai Co., Ltd.). The target gene expression values were normalized to those of β-actin. The primers used for RT-PCR are shown in Table 1, and the 2^−ΔΔCt^ method was used for data analysis.

**Table 1:** Sequences of the primers used in the RT-PCR experiments

Gene	Sequence (5′ to3′)
BMP2-F	AGCGGAAACGCCTCAAAT
BMP2-R	ACGATGGCATGGTTAGTGGA
OPG-F	AGGAGTTGCAGCACGTCAAG
OPG-R	CTGTATTCCGCTCTGGGGTT
RANKL-F	CCACCAACATCCCATCAGG
RANKL-R	GCGTACAGGTAATAAAAGCCATC
β-actin-F	TGGCACCCAGCACAATGAA
β-actin-R	CTAAGTCATAGTCCGCCTAGAAGCA

### Western blot

Western blot analysis was used to assess the expression of osteogenesis-related proteins (BMP2, OPG, and RANKL) in the three experimental groups. Bone callus fragments were finely diced on ice and then added to a cell lysate composed of radioimmunoprecipitation assay (RIPA) buffer (Beyotime Biotechnology, Shanghai, China; catalog number: P0013B) supplemented with 2% protease inhibitor (APPLYGEN, Beijing, China; catalog number: P1265). A small amount of liquid nitrogen homogenate was subsequently added, and after allowing to settle, the protein lysates were collected by centrifugation (12 × g, 4 °C, 20 min) and quantified using a BCA kit (ApplyGEN, China; list number: P1511). Following denaturation in a boiling water bath, the proteins were separated by 10% sodium dodecyl sulfate-polyacrylamide gel electrophoresis (SDS-PAGE) and subsequently transferred to a polyvinylidene difluoride (PVDF) membrane. After being blocked with 5% nonfat milk for 1 h and washed three times with Tris-buffered saline with Tween 20 (TBST), the membrane was incubated with the primary antibodies at 4 °C overnight (anti-BMP2: Bioss, Beijing, China; catalog number: bs-1012R; anti-OPG: Bioss; catalog number: bs-20624R; anti-RANKL: Bioss; catalog number: bs-20647; anti-GAPDH: Bioss; catalog number: bsm-33033 M). Following three additional washes with TBST, the membrane was incubated with a horseradish peroxidase secondary antibody (1:500; Beijing Conway Century Biotechnology Co., Ltd., Beijing, China; catalog number: CW0103) for 1 h. Finally, the bands were detected using a chemiluminescence imaging system (Bio-Rad).

### Statistical analysis

The data are presented as the means ± standard deviations. Statistical analyses were performed using Prism 8.3.0 software (GraphPad, La Jolla, CA, USA). For normally distributed data, Student’s *t* test was used for comparisons between two groups, and one-way analysis of variance was used for comparisons between multiple groups. Nonnormally distributed data were subjected to nonparametric tests, with the Mann‒Whitney *U* test employed for comparisons between two groups and the Wilcoxon test used for comparisons among multiple groups. Additionally, the chi-square test was applied for comparisons involving count data between two groups. Finally, *p* < 0.05 was considered statistically significant.

## Results

### Axial dynamization of MVFP in vitro and in vivo

**Fig. 2:**
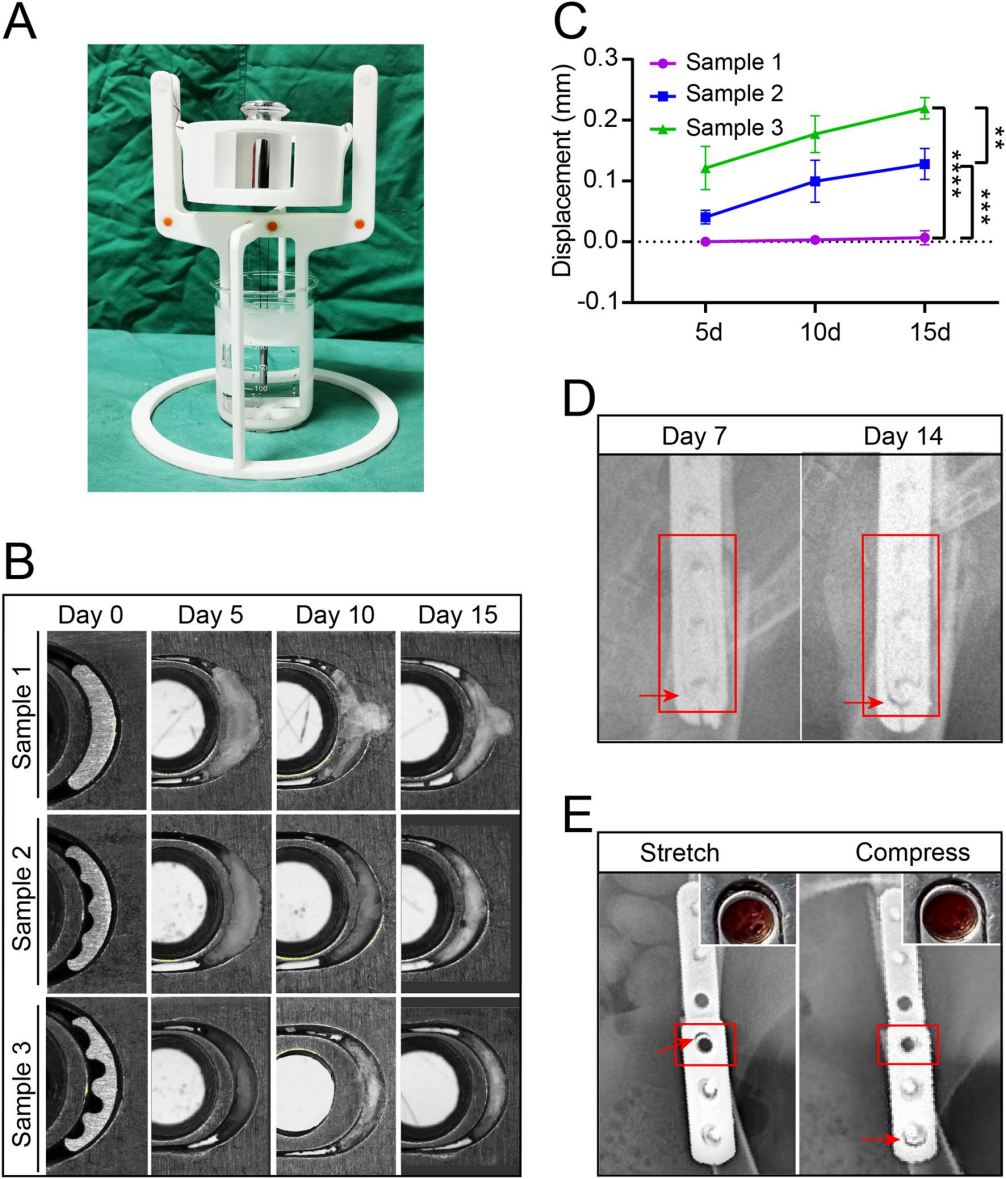
Dynamic transformation of MVFP in vitro and in vivo. **A** In vitro simulation of a support system in which 3 kg of pressure is continuously applied to the slider and shim using a pulley system. **B** Degradation of three types of shims in vitro under continuous pressure of 3 kg. **C** Displacement of the MVFP slider at different time points under a continuous pressure of 3 kg in an in vitro dynamic transformation experiment. **D** Dynamic transformation of MVFP in animals observed by X-ray photography. **E** The dynamic status of the MVFP was confirmed through X-ray photography and dissection. Significance level: ns, no significant difference; *, *p* < 0.05; **, *p* < 0.01; ***, *p* < 0.001; ****, *p* < 0.0001

The specially designed stand employed pulleys to transmit the gravitational force of the weight onto the slider, thereby generating a 3 kg pressure on the shim (Fig. 2A, B). The average weights of the three types of shims were 2.76 ± 0.077 mg/piece, 2.86 ± 0.032 mg/piece, and 2.39 mg ± 0.093 mg/piece. Among the three types of shims, sample 3 produced the greatest mean axial displacement in vitro under continuous pressure equivalent to one body weight of the rabbit, which was 0.121 mm, 0.177 mm, and 0.220 mm at 5, 10, and 15 days of immersion, respectively  (Fig. 2C, D). The dynamization of the plate in vivo was monitored through X-ray imaging. At 7 days postimplantation, the gap between the end side of the plate and the slider appeared small and indistinct, with limited axial dynamization observed in the slider. However, at 14 days postimplantation, the gap between the slider and the plate became clearly visible, indicating successful dynamization of the plate within 2 weeks (Fig. 2E). After 2 weeks in vivo, the shim (Sample 3) underwent significant transformation, predominantly converting into black metal debris (Fig. 2F). Residues were found adhering to both the plate and the slider, allowing free axial sliding along the plate. In summary, MVFP with a shim (sample 3) enabled the slider to gradually achieve more than 0.2 mm of axial micromotion approximately 2 weeks after the initial rigid fixation.

### Radiographic evaluation of MVFP in a rabbit femur fracture model

**Fig. 3:**
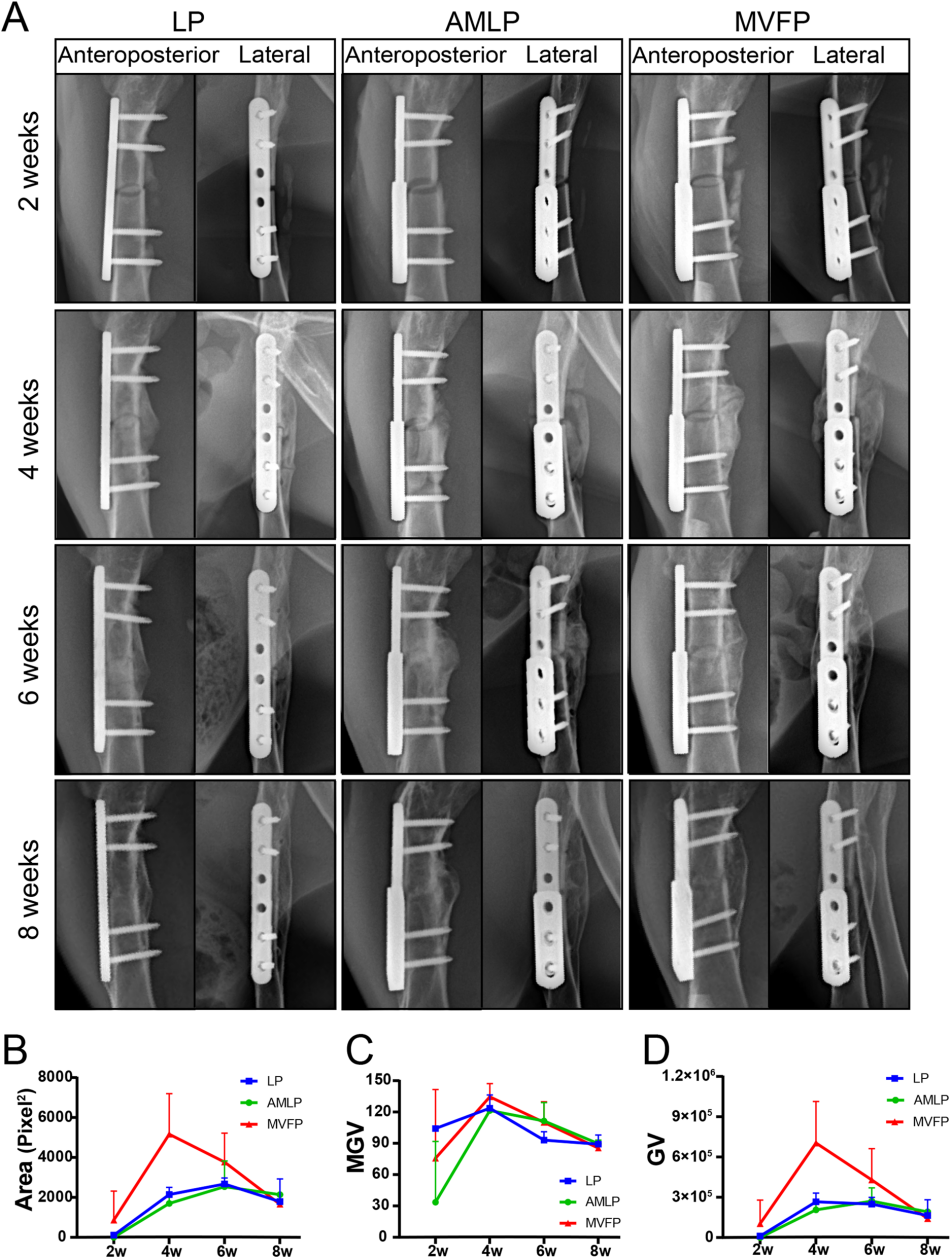
Serial X-ray images of the affected limbs in the LP, AMLP and MVFP groups after surgery. **A** Anteroposterior and lateral femoral X-ray images of the affected limbs of the three groups at 2, 4, 6, and 8 weeks after the operation. Scatter plots of the projected callus area (**B**), average gray value (**C**), and total gray value (**D**) on anteroposterior femoral radiographs of the three groups after surgery (*n* = 9)

Continuous observation of fracture healing via X-ray films from 2 to 8 weeks revealed that the callus volume was minimal at 2 weeks postoperation, exhibited a substantial increase by the 4th week, and then gradually decreased between the 6th and 8th weeks postoperation (Fig. 3A). Complete healing of the fracture was observed by the 8th week postoperation. Radiographically, the MVFP group displayed greater and denser callus formation than the other two groups did.

Quantitative calculations of the callus projections on AP radiographs suggest that the callus areas in the LP and AMLP groups still increased at 4 weeks postimplantation and peaked at 6 weeks postimplantation, whereas in the MVFP group, the callus area peaked at approximately 4 weeks postimplantation (Fig. 3B). The MGV across all the groups reached its peak at 4 weeks after the operation, with the MVFP group achieving the highest peak value (Fig. 3C). The total GVs for both the MVFP and LP groups peaked at approximately 4 weeks after surgery, whereas the AMVP group reached its maximum at 6 weeks (Fig. 3D). Comparative analysis of the callus area, MGV, and TGV among the three groups at 4 weeks postsurgery revealed statistically significant differences only in the callus area and TGV between the AMLP and MVFP groups.

### Micro-CT evaluation of MVFP in a rabbit femur fracture model

**Fig. 4:**
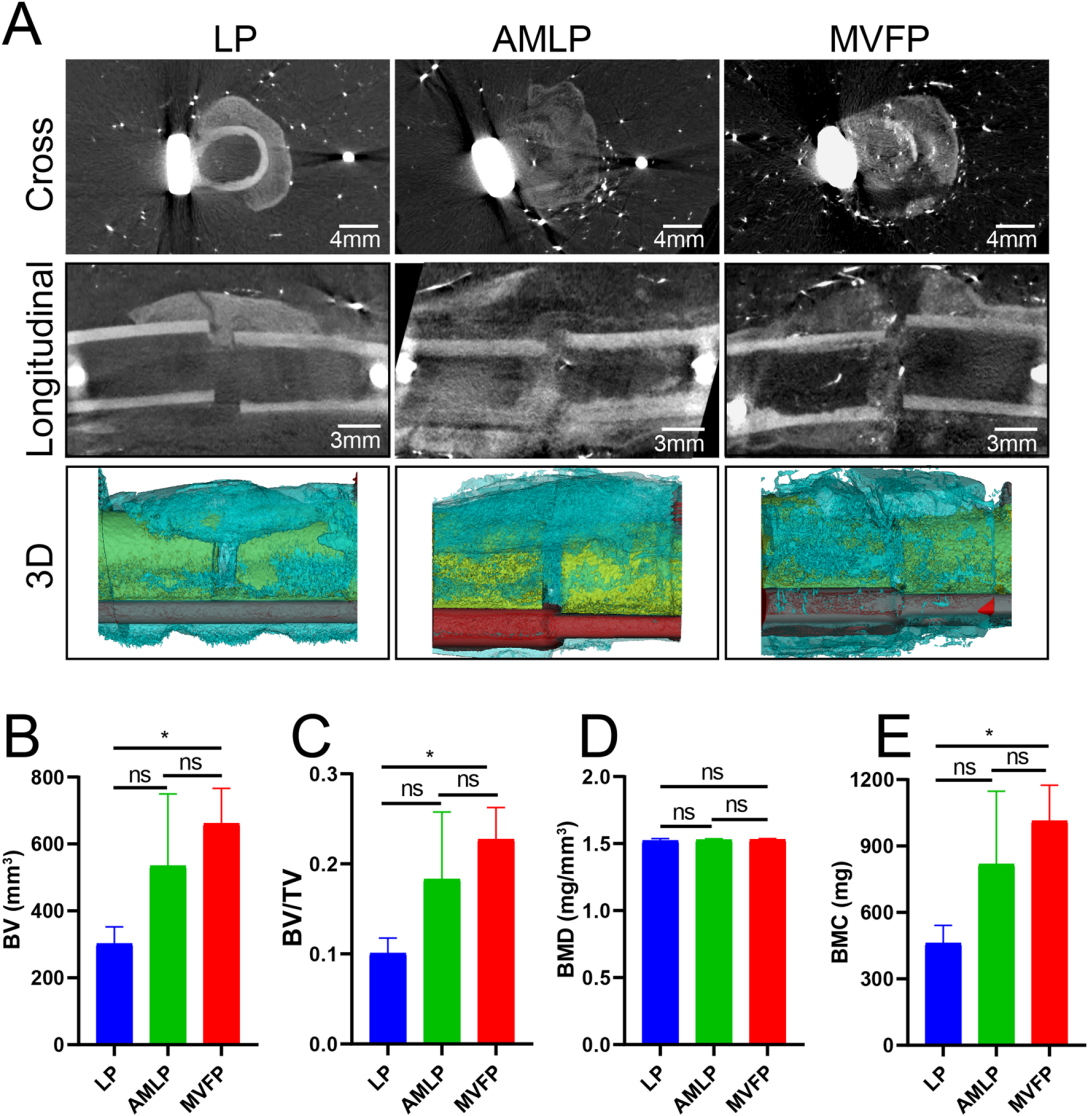
Evaluation of the healing of femoral fractures in the three groups by micro-CT at 4 weeks after operation. **A** Cross-sectional, longitudinal and three-dimensional micro-CT images of the femoral fracture area on the affected side. Bar plots of new bone volume (**B**), bone volume fraction (**C**), bone mineral density (**D**), and bone mineral content (**E**) in the region of interest (*n* = 9). Significance level: ns, no significant difference; *, *p* < 0.05

At 4 weeks postimplantation, both transverse and longitudinal micro-CT scans revealed that the MVFP group presented the greatest quantity of new calli, followed by the AMLP group, and the LP group presented the least formation of new calli (Fig. 4A). Three-dimensional CT reconstruction and quantitative assessment of new bone volume revealed that the MVFP group presented the greatest volume of new bone, which significantly differed from that of the LP group (Fig. 4B). Although the mean callus volume in the AMLP group was greater than that in the LP group, the substantial standard deviation resulted in a statistically insignificant difference between the two groups. The bone volume fraction, in line with the trend observed in new bone volume, was significantly different between the MVFP group and the LP group in pairwise comparisons among the three groups (Fig. 4C). Notably, there were no significant differences in the mean BMD among the three groups (Fig. 4D). However, the disparity in bone mineral content (BMC) among the groups corresponded with the differences in bone volume (BMC = BMD × total volume), illustrating similar trends (Fig. 4E).

**Fig. 5:**
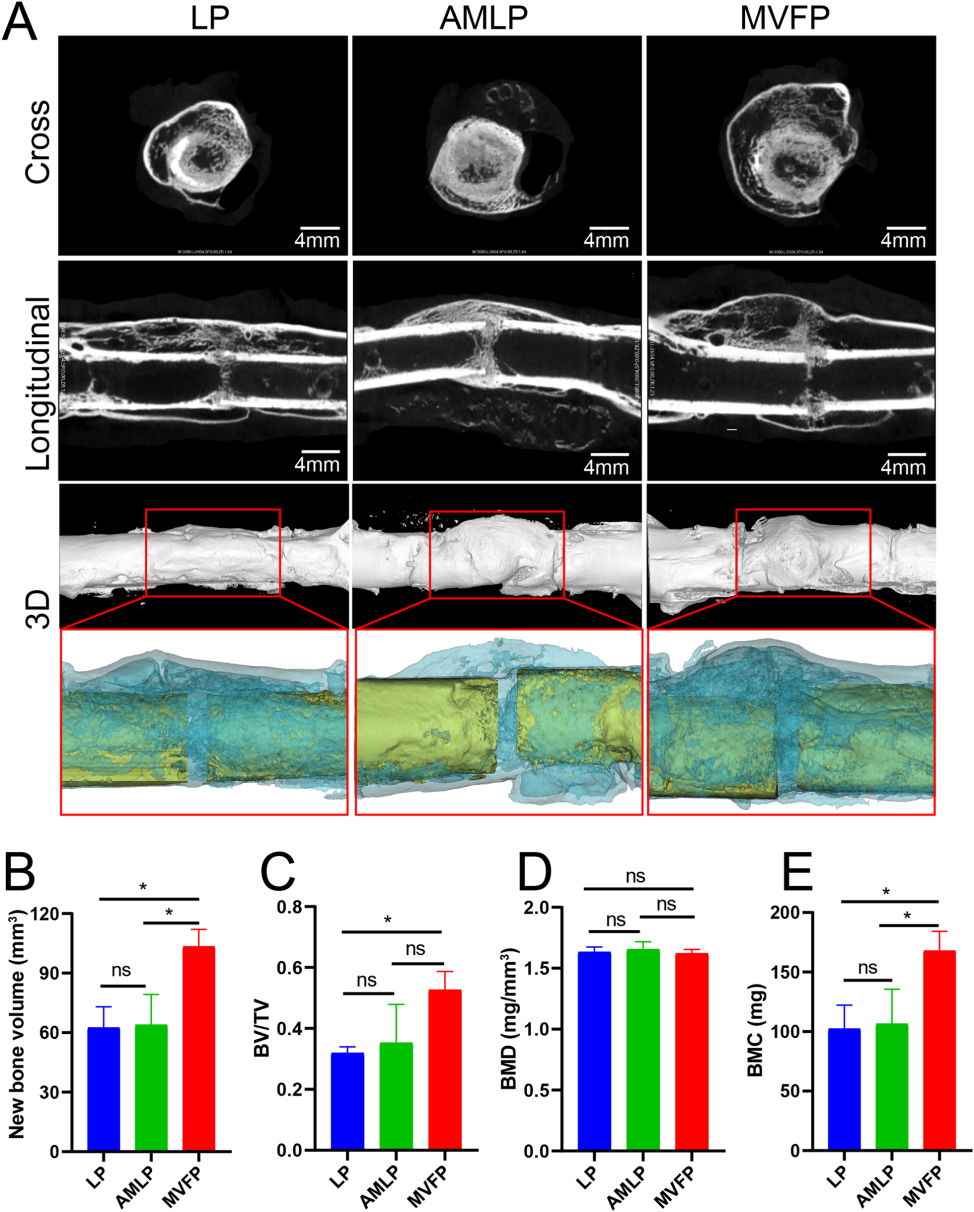
Evaluation of femoral fracture healing in the three groups by micro-CT at 8 weeks after the operation. **A** Cross-sectional, longitudinal and three-dimensional micro-CT images of the femoral fracture area on the affected side. Bar plots of new bone volume (**B**), bone volume fraction (**C**), bone mineral density (**D**), and bone mineral content (**E**) in the region of interest (*n* = 9). Significance level: ns, no significant difference; *, *p* < 0.05

At 8 weeks postimplantation, both cross-sectional and longitudinal micro-CT scans revealed a greater presence of new bone in the MVFP group than in the other two groups (Fig. 5A). Notably, the callus volume at 8 weeks was smaller than that at 4 weeks, potentially owing to bone remodeling. Computer 3D reconstruction revealed greater new bone volume in the MVFP group than in the other two groups, whereas no significant difference was noted between the LP and AMLP groups (Fig. 5A, B). The new bone volume fraction was greater in the MVFP group than in the other two groups, although no significant difference was observed between the AMLP and MVFP groups (Fig. 5C). Additionally, there was no significant difference in the bone volume fraction between the LP and AMLP groups. The mean BMD of the region of interest was comparable and was not significantly different (Fig. 5D). Consequently, this similarity in BMD contributed to similar BMC and new bone volume, indicating that the BMC in the MVFP group was significantly greater than those in the other two groups (Fig. 5E).

### Toluidine blue staining of the calli in different groups

**Fig. 6:**
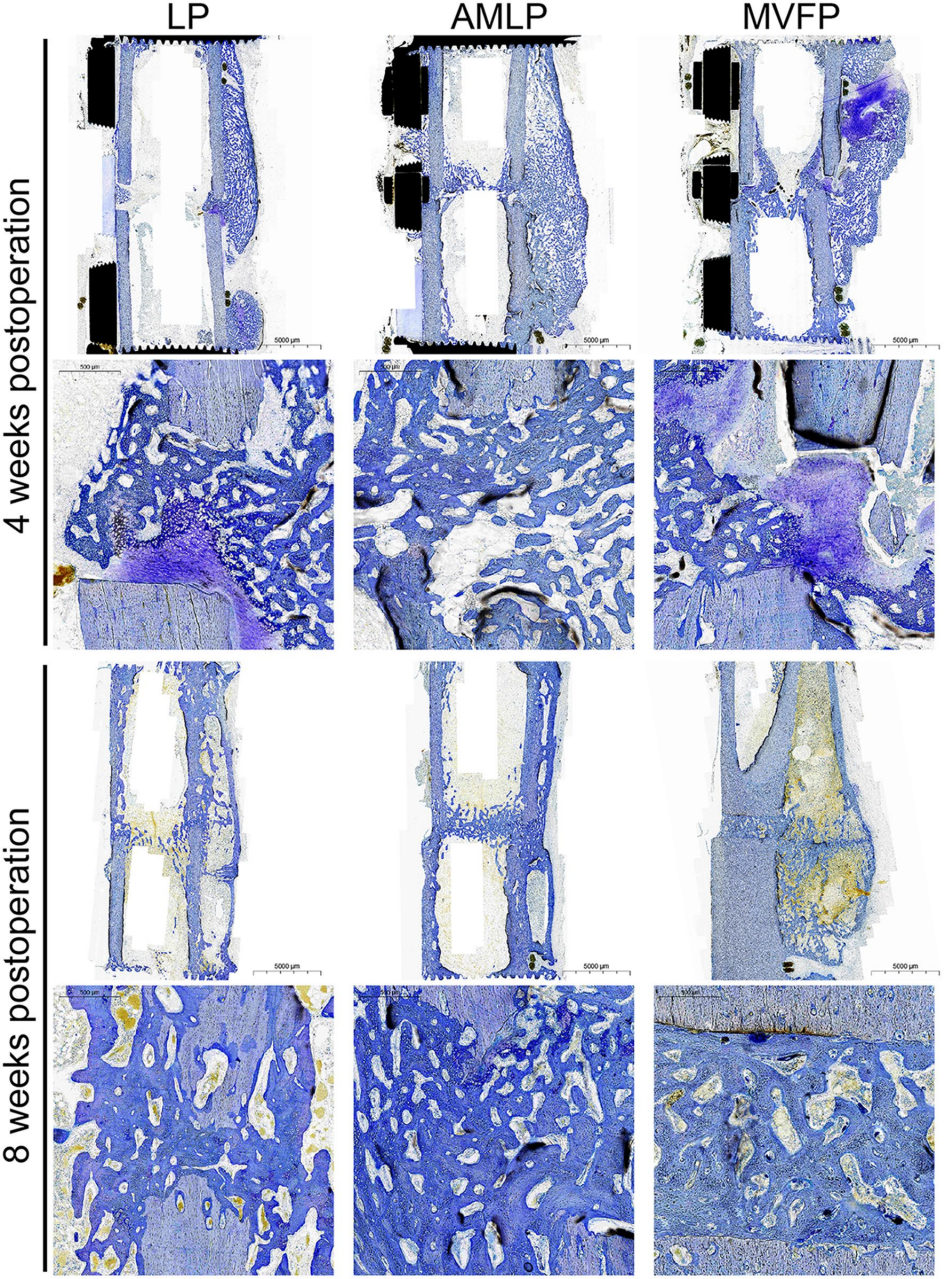
Toluidine blue staining of tissues at the fracture sites at 4 and 8 weeks postoperation (general view and 30 × magnification)

Toluidine blue staining of bone healing tissue at 4 weeks postoperation revealed increased callus formation at fracture ends in the AMLP and MVFP groups compared with the LP group, with denser trabecular bone (Fig. 6). The calluses filled the gaps between the fracture ends adjacent to the plate in both experimental groups, in contrast with the LP group, where the gap remained visible and callus was sparse.

At 8 weeks postoperation, the callus remained sparse in the LCP group but was increased in size compared with that at 4 weeks. The MVFP group presented lighter-colored callus between fracture ends, suggesting less cartilage tissue and more mature bone formation, whereas the AMLP group presented darker callus, indicating more cartilage and less mature bone. All groups showed bony junction formation at the fracture ends by 8 weeks, which was consistent with previous imaging findings. Notably, there were more intraosseous calli on the plate side in both the AMLP and MVFP groups. In contrast, there was a markedly lower presence of intraosseous callus on the plate side in the LP group than in the control group, which might have led to asymmetric osteogenesis.

### Masson’s trichrome staining

**Fig. 7:**
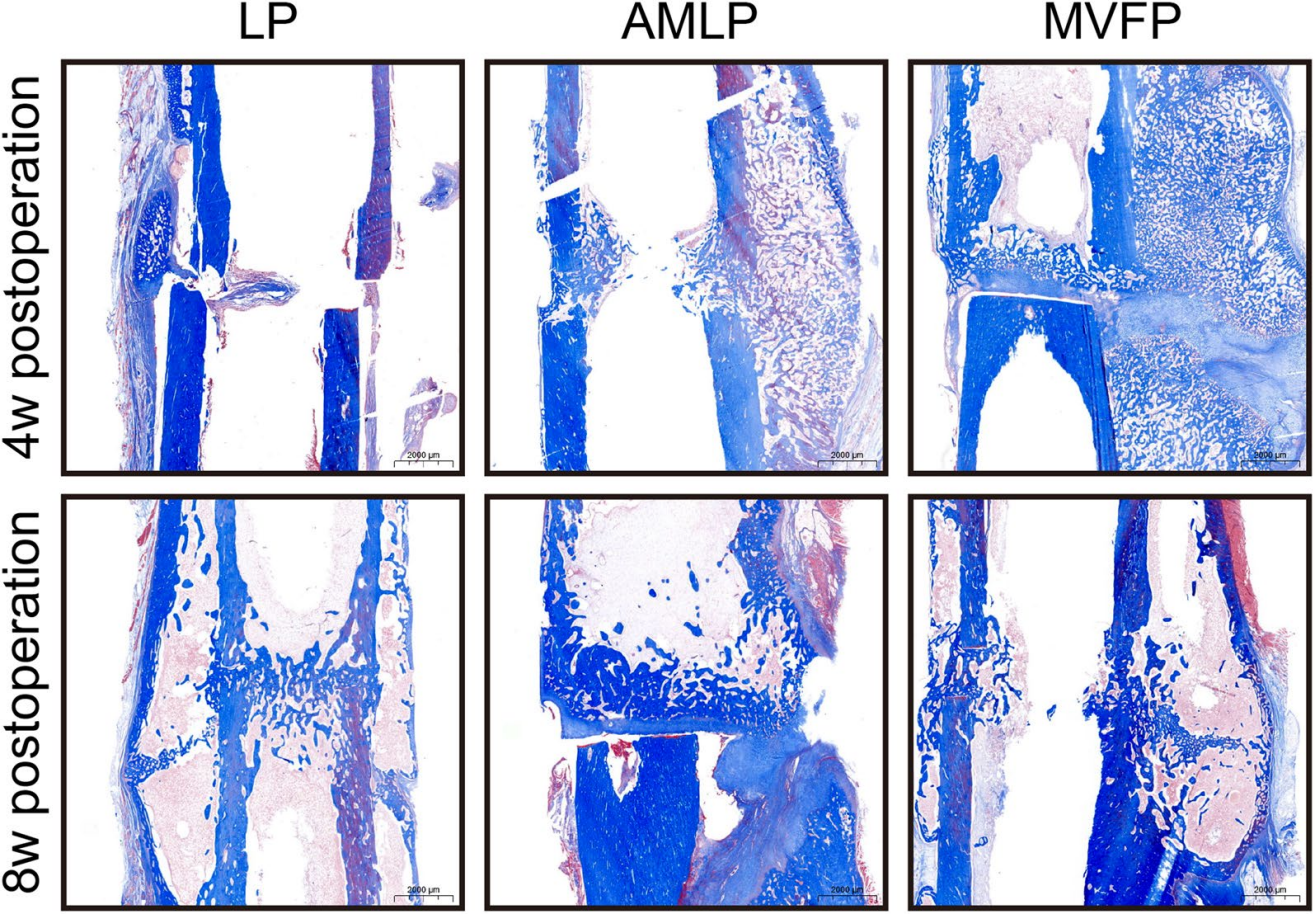
Masson’s trichrome staining results of bone healing tissue at 4 and 8 weeks postoperation

Masson’s trichrome staining revealed notable differences among the three groups. Specifically, at 4 weeks postoperation, the LP group presented the least amount of callus formation at the fracture ends, with minimal callus formation observed on the plate side. Conversely, the MVFP group demonstrated substantial callus formation, with the callus extending toward the contralateral side and displaying a dense trabecular bone density within the callus area. In comparison, the extraosseous calli in the AMLP group contained more nonosseous components (red-stained areas) and fewer osseous components compared with those in the MVFP group (Fig. 7).

By the 8-week mark postoperation, all groups had formed a dense bone junction between the fracture ends, with the original callus being reabsorbed and reconstructed, resulting in a dense cavity at the edge. Notably, the callus on the LP side appeared sparser, and the cortical bone displayed more nonosseous components (red staining area) than did those on the contralateral side in the LP and MVFP groups. These findings suggest that the bone healing quality of the LP group was inferior to those of the other groups. Importantly, these findings align with those obtained from toluidine blue staining, X-ray, and micro-CT analyses.

### Torsion test of the healed femurs

**Fig. 8:**
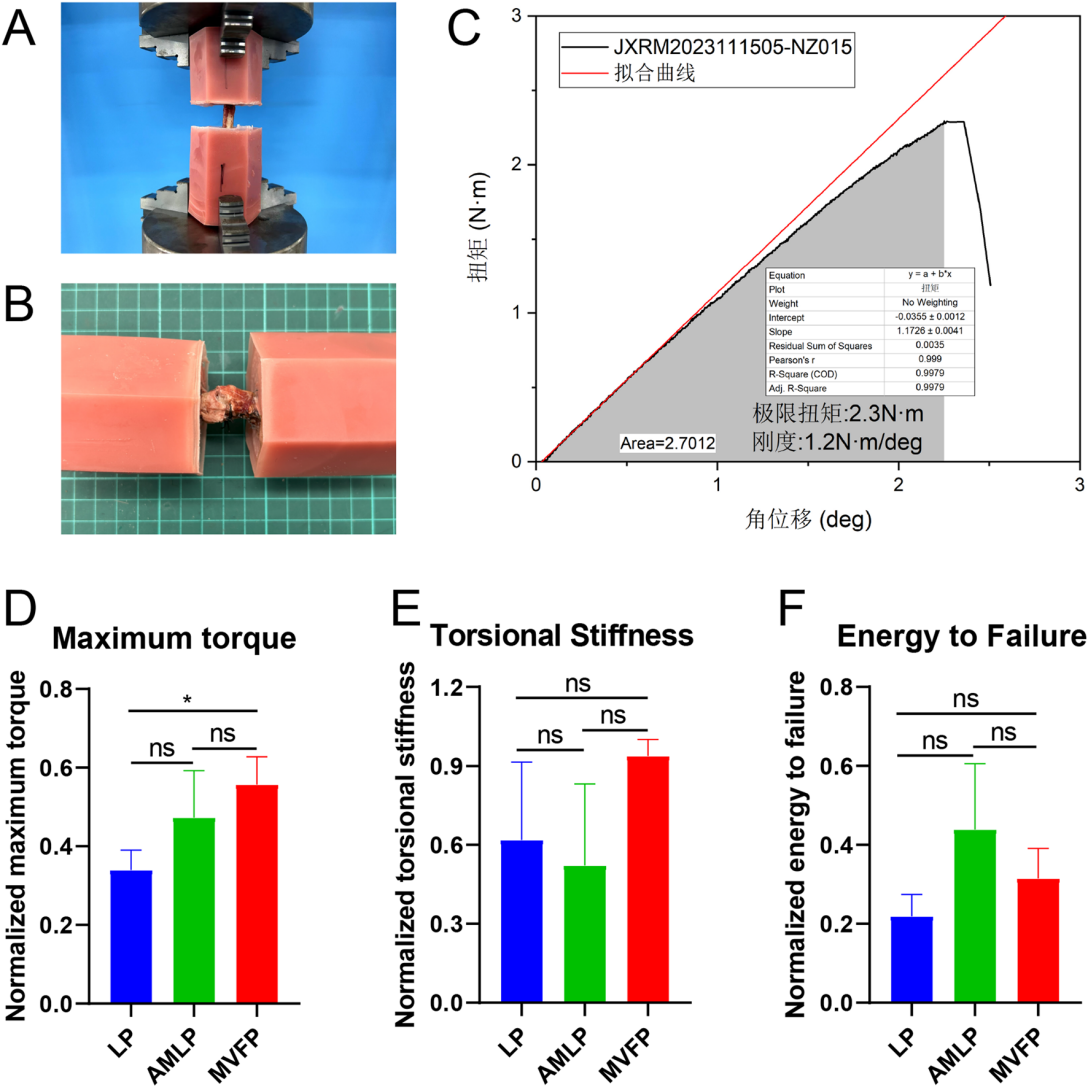
Torsion test of the healed bone in the LP, AMLP, and MVFP groups. **A** The presentation of a healed bone in the torsion test. **B** Morphology of refractures after the torsion test. **C** Output of the results of the ultimate torque, stiffness and area under the curve of the torsion test. Bar plots of the maximum torque (**D**), torsional stiffness (**E**), and failure energy (**F**) of the three groups of samples (*n* = 9). Significance level: ns, no significant difference; *, *p* < 0.05

The torsion test conducted at 8 weeks postoperation revealed that the LP group exhibited the lowest relative maximum torque among the healed femurs, approximately 34% of that of the healthy femur (Fig. 8A–D). The AMLP group presented approximately 47% of that of the healthy femur, and the MVFP group presented the highest torque at approximately 56% of that of the healthy femur. The MVFP group’s relative maximum torque was significantly greater than that of the LP group, but no significant difference was detected between either the LP and AMLP groups or the AMLP and MVFP groups.

In terms of torsional stiffness, the average relative stiffness of the healed femurs was the lowest in the AMLP group, approximately 52% of that of the healthy femur (Fig. 8E). The LP group ranked second, with approximately 62% of that of the healthy femur, whereas the MVFP group had the highest stiffness at approximately 94% of that of the healthy femur. Despite the AMLP group having the greatest relative damage energy, there was a large within-group difference, and no statistically significant difference was found between any two of the three groups (Fig. 8F).

In summary, at 8 weeks postoperation, the MVFP group demonstrated greater average relative maximum torque, stiffness, and failure energy than did the LP group. The AMLP group also had a higher relative maximum torque compared with that of the LP group, but its torsional stiffness was not as effective as those of the LP and MVFP groups. This may be attributed to delayed bone mineralization and remodeling in the AMLP group, as observed in postoperative radiographs. The AMLP group featured more incompletely mineralized calli, resulting in lower relative stiffness but greater toughness and, consequently, greater relative failure energy.

Ultimately, the maximum torque, stiffness, and destructive energy of the healed femur remained lower than those of the healthy femur at 8 weeks postoperation. This finding suggested that although bony connections had formed at the fracture ends, the healing strength was still insufficient, and more time was needed to reach the level of a healthy femur.

### mRNA expression profiles of osteogenesis-related genes

**Fig. 9:**
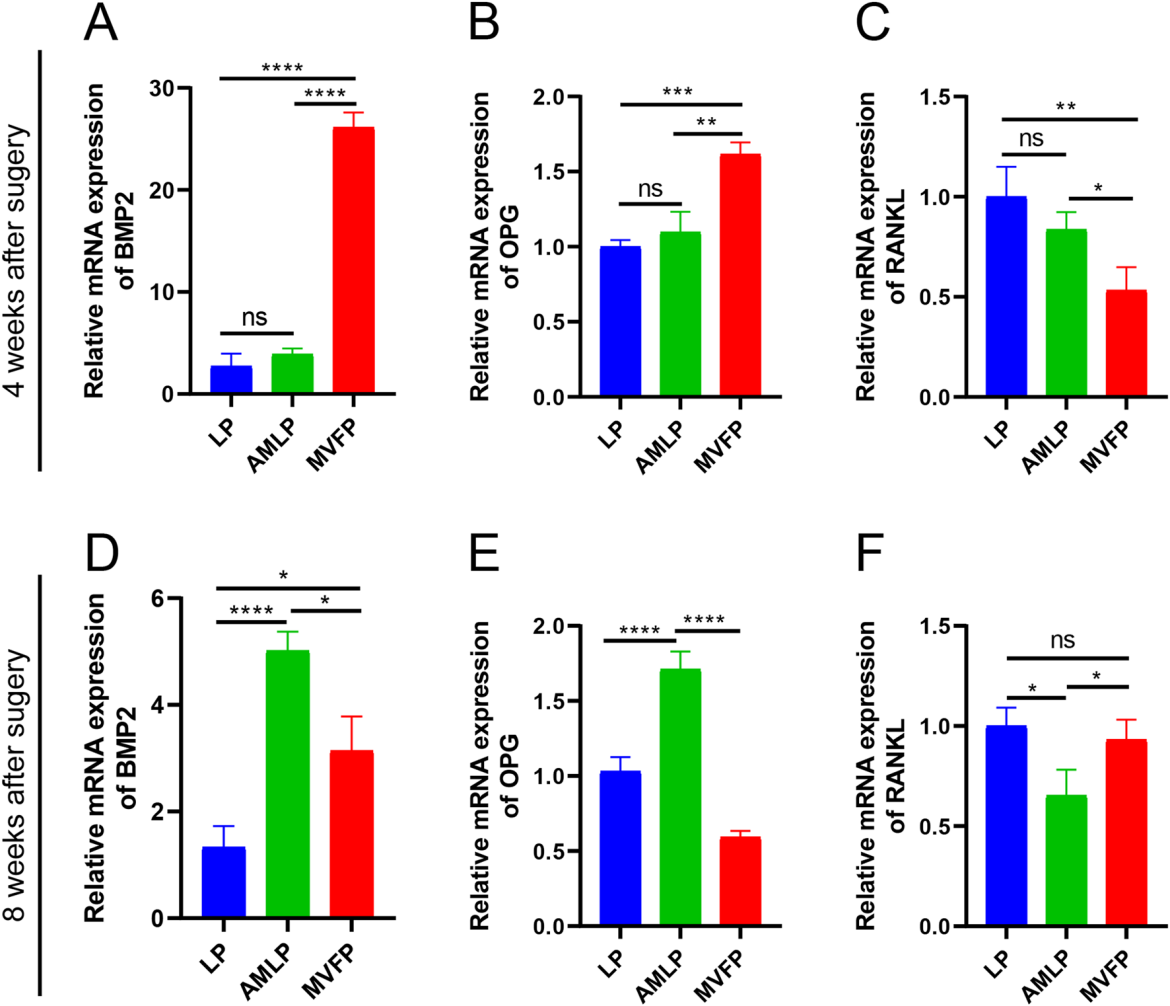
Relative expression levels of osteogenesis-related genes in the LP, AMLP and MVFP groups determined by RT-PCR. Relative expression levels of BMP2 (**A**), OPG (**B**), and RANKL (**C**) 4 weeks after surgery and relative expression levels of BMP2 (**D**), OPG (**E**), and RANKL (**F**) 8 weeks after surgery (relative to β-actin). Significance level: ns, no significant difference; *, *p* < 0.05; **, *p* < 0.01; ***, *p* < 0.001; ****, *p* < 0.0001

At 4 weeks postoperation, the mRNA expression levels of BMP2 and OPG were significantly greater in the MVFP group than in the other groups (Fig. 9A, B), whereas RANKL expression was notably lower in the MVFP group than in the other groups (Fig. 9C). Although no significant differences in BMP2, OPG, or RANKL messenger RNA (mRNA) expression were detected between the LP and AMLP groups, the AMLP group exhibited slightly higher BMP2 and OPG expression and slightly lower RANKL expression compared with the LP group.

At 8 weeks postoperation, BMP2 expression remained significantly greater in both the AMLP and MVFP groups than in the LP group, with the AMLP group showing the highest expression (Fig. 9D). Osteogenic activity was greater in the AMLP and MVFP groups than in the LP group, with the AMLP group showing the highest activity, which differed from the 4-week results. OPG mRNA expression was highest in the AMLP group, followed by the LP group, and was lowest in the MVFP group (Fig. 9E). RANKL expression was lowest in the AMLP group and highest in the LP group, with the expression level in the MVFP group similar to that in the LP group (Fig. 9F). Compared with that in the LP group, RANKL expression was higher in the MVFP group at 4 weeks, indicating similar osteoclast activity, possibly increasing over time owing to bone remodeling in the later stages of healing.

### Protein expression levels of osteogenesis-related genes

**Fig. 10:**
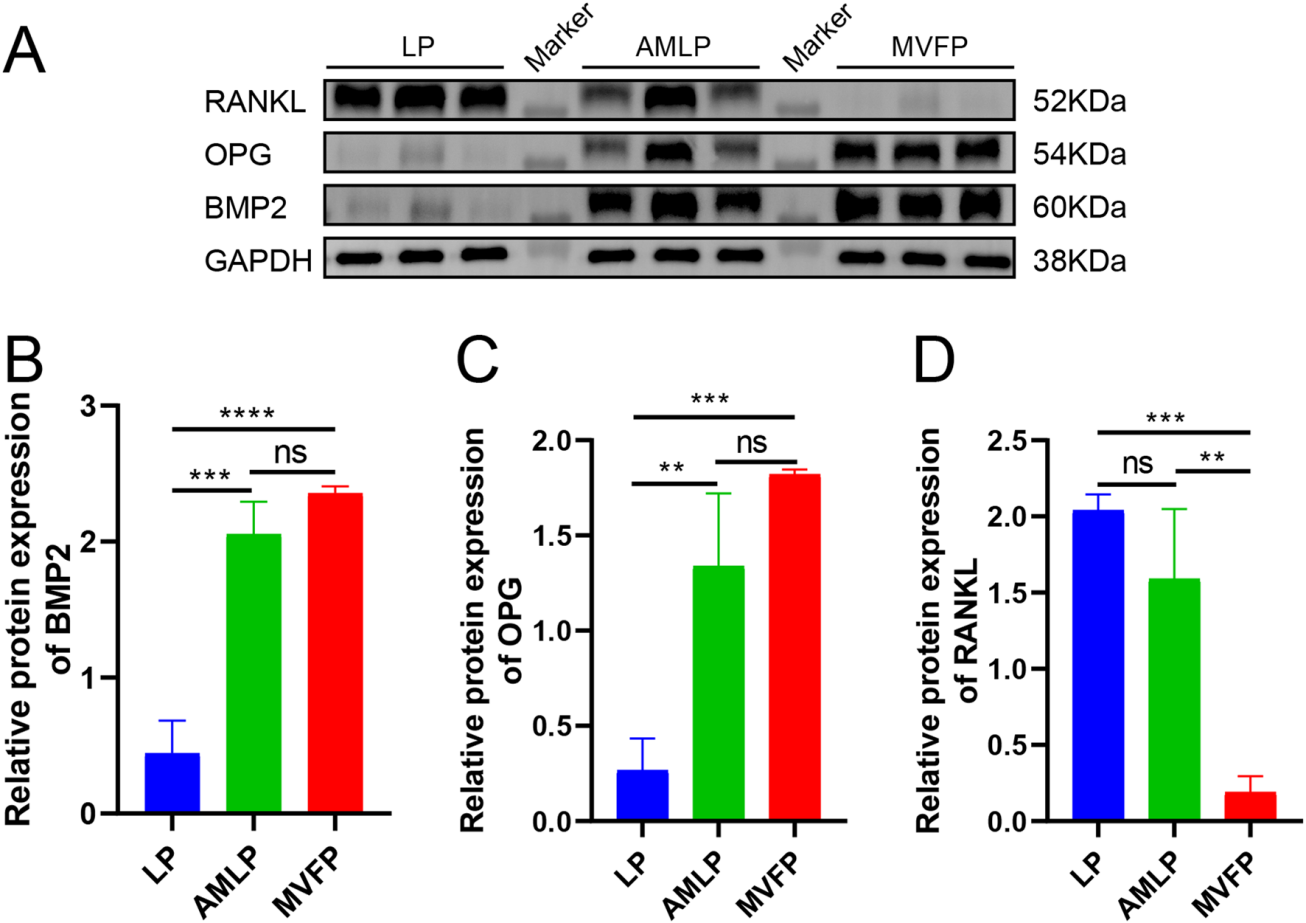
Relative protein expression levels of osteogenesis-related genes in the LP, AMLP and MVFP groups according to western blotting at 4 weeks after surgery. **A** Western blot analysis of the protein expression levels of BMP2, OPG, and RANKL in the three groups at 4 weeks after surgery. Relative expression levels of BMP2 (**B**), OPG (**C**), and RANKL (**D**) normalized to those of GAPDH (internal reference) (*n* = 9). Significance level: ns, no significant difference; *, *p* < 0.05; **, *p* < 0.01; ***, *p* < 0.001; ****, *p* < 0.0001

At 4 weeks postoperation, Western blot analysis of bone calli revealed significantly higher protein expression levels of BMP2 and OPG in the AMLP and MVFP groups than in the LP group (Fig. 10A–C). Although these proteins were slightly more highly expressed in the MVFP group than in the AMLP group, the difference was not statistically significant. Additionally, RANKL protein expression was significantly lower in the MVFP group than in the other two groups, with no notable difference between the LP and AMLP groups (Fig. 10A, D). Osteogenesis-related gene expression was significantly increased in the AMLP and MVFP groups but was lowest in the LP group at this time point. The high expression of OPG and low expression of RANKL in the MVFP group suggested lower osteoclast activity than in the other groups.

**Fig. 11:**
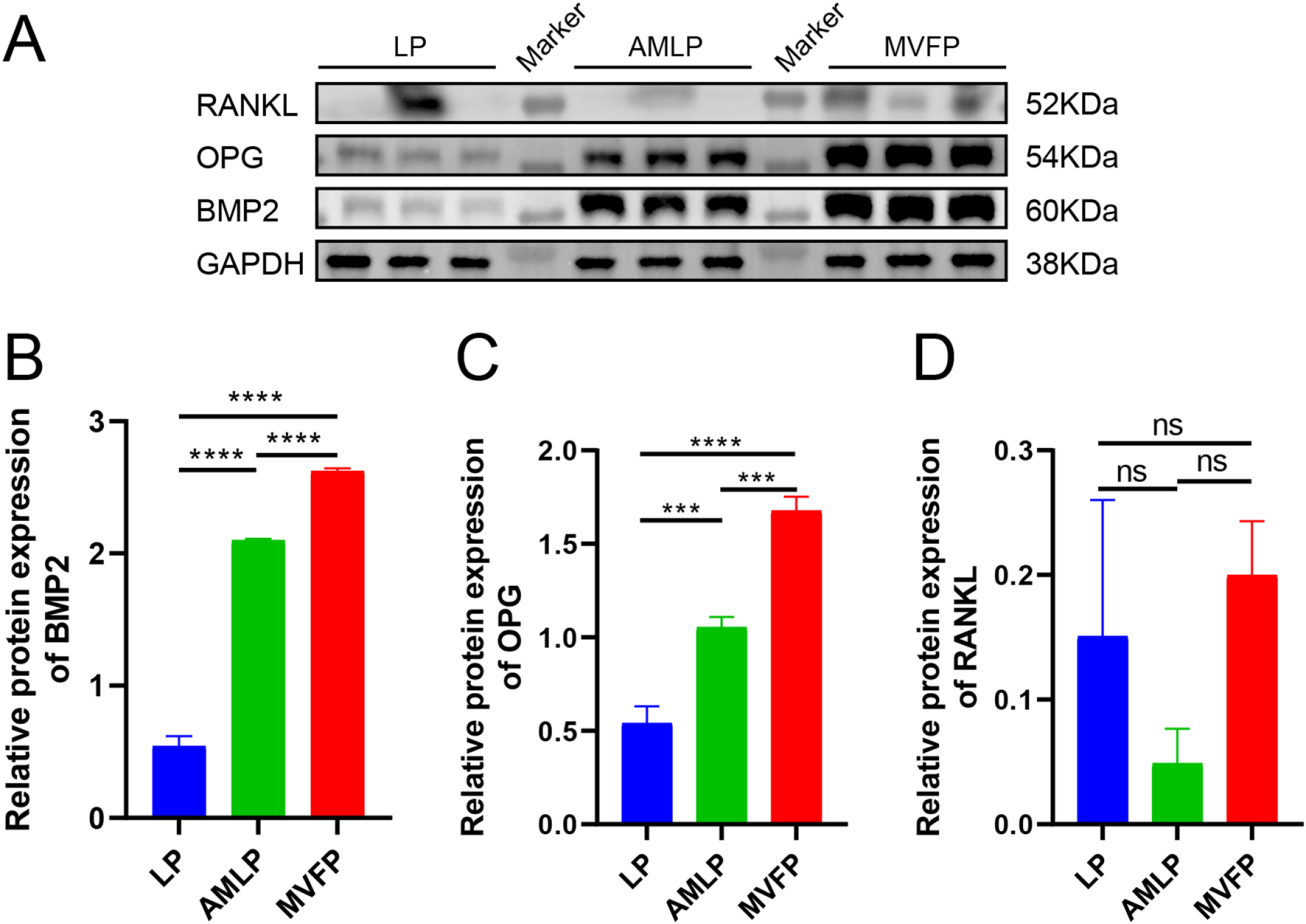
Relative protein expression levels of osteogenesis-related genes in the LP, AMLP and MVFP groups according to western blotting 8 weeks after surgery. **A** Western blot analysis of the BMP2, OPG, and RANKL proteins in the three groups 8 weeks after surgery. Relative expression levels of BMP2 (**B**), OPG (**C**), and RANKL (**D**) normalized to those of GAPDH (internal reference) (*n* = 9). Significance level: ns, no significant difference; *, *p* < 0.05; **, *p* < 0.01; ***, *p* < 0.001; ****, *p* < 0.0001

At 8 weeks postoperation, western blot analysis revealed greater BMP2 protein expression in the AMLP and MVFP groups than in the LP group, indicating active osteogenesis in these groups (Fig. 11A, B). However, OPG expression was lowest in the MVFP group, whereas RANKL expression was highest, indicating increased osteoclast activity in the MVFP group, thereby promoting bone resorption and remodeling (Fig. 11A, C, D). Conversely, the AMLP group, which presented the lowest RANKL expression, exhibited increased bone activity and reduced osteoclast activity, suggesting an early to middle stage of bone healing characterized by increased active osteogenesis.

## Discussion

With advancements in plate technology, LPs have gained widespread acceptance in clinical practice. Unlike conventional plates, LPs prioritize biological fixation for fracture healing through the use of titanium alloy material, which reduces the stiffness of internal fixation, with a focus on promoting fracture healing through callus formation [27–29]. An increasing number of studies have demonstrated that axial micromotion within the range of 0.2–1 mm at fracture ends is beneficial for stimulating callus formation and facilitating bone healing. The findings of this study align with literature, as the mean callus volume of the AMLP group was greater than that of the LP group [13–18].

With respect to the timing of micromotion, most scholars agree that maintaining stability at the fracture ends during the early stages of healing is crucial for optimal fracture recovery. However, there is inconsistency in literature regarding the specific definition of the “early stage.” Nonetheless, most researchers concur that providing mechanical stability to fracture ends for a period ranging from 4–8 days after fracture is beneficial for promoting osteoblast differentiation and angiogenesis [20–24]. On the basis of this knowledge, shims are suggested to be able to maintain integrity for approximately 4–8 days. In this study, the duration of axial dynamization was approximately 7–14 days, which is similar to the time reported in literature. This timeframe aligns effectively with the crucial period of hematoma organization and angiogenesis that occurs after a fracture [30, 31]. After 7–14 days of rigid fixation, the plate automatically transitions to limited axial micromotion fixation, which aims to stimulate callus formation and enhance bone healing by striking a balance between initial stability and subsequent micromotion.

The shims utilized in this particular study were made of pure magnesium metal and had a mass of approximately 2.39 mg per shim. Importantly, magnesium is a common element found in the human body and ranks fourth among cations. An average healthy adult typically stores between 24 and 30 g of magnesium in their body and requires an additional 310–420 mg of magnesium per day to maintain normal physiological functions [32, 33]. Therefore, the dosage of 2.39 mg of magnesium used in this study does not pose a risk of excessive poisoning in humans. The degradation of magnesium in the body can potentially generate magnesium hydroxide and hydrogen gas [34]. The complete degradation of 2.39 mg of magnesium from a shim would theoretically yield approximately 9.83 × 10^−5^ mol of magnesium hydroxide and release approximately 2 ml of hydrogen gas (under standard atmospheric pressure). These byproducts may have a certain impact on the local pH value. Importantly, however, mammals possess a relatively robust buffer system, which helps maintain homeostasis. Additionally, magnesium degrades at a very slow rate in vivo (average corrosion rate in vivo: 0.63 mm/yr) [35, 36], which further mitigates any potential effects of the small amount of magnesium hydroxide produced. Consequently, the local effect of these byproducts, including small amounts of hydrogen, is minimal and unlikely to have a significant impact. Notably, in this study, there were no indications of obvious subcutaneous gas formation surrounding the plate in the MVFP group; this suggests that the small amount of hydrogen generated through magnesium degradation did not lead to the creation of noticeable gas pockets in the surrounding tissue. In summary, the dosage of magnesium used in this study was within a safe range, and any byproducts generated from magnesium degradation had minimal effects owing to the body’s buffer system and the slow degradation rate of magnesium in vivo. The absence of subcutaneous gas around the plate further supports the lack of significant local effects. Our findings regarding the degradation properties of the magnesium shim align closely with those reported by Patrick K. Bowen et al. [37].

X-ray and CT scans revealed that the new callus volume was most pronounced in the MVFP group, followed by the AMLP group and then the LP group. Animal experiments revealed that MVFP notably excelled in fostering callus formation and expediting bone healing. Although the AMLP group presented greater callus volume than the LP group did in this study, notable individual differences were observed, emphasizing the need for further validation through a larger sample size experiment. Nonetheless, an increasing body of research suggests that AMLP significantly contributes to fracture healing [13–18], with some studies reporting promising outcomes in clinical research [17–19, 38]. Moreover, our investigation revealed no significant variance in the mean BMD across the three groups. These findings suggest that although axial micromotion might have a limited effect on BMD, it could facilitate callus formation. Toluidine blue and Masson’s trichrome staining corroborated these findings, revealing a greater volume of new callus formation within the MVFP group, which was consistent with observations from X-ray and CT scans. Moreover, strong fixation on the plate side might impede the formation of calli. Additionally, the outcomes of the torsion test offered further validation, highlighting greater rigidity in the healed bones of the MVFP group than in those of the remaining two groups. This improved stiffness can be attributed, in part, to the increased callus formation observed specifically in the MVFP group. These collective findings emphasize the possible influence of axial micromotion on both callus formation and the resulting mechanical properties of healed bones.

In this study, we detected the expression of three groups of osteogenesis-related genes at different time points. MVFP may enhance osteoblast differentiation and function by increasing BMP2 expression [39–41] and regulating the OPG/RANKL/RANK signaling axis to influence osteoclast differentiation and activity [42–44]. This dual mechanism potentially accelerates and strengthens fracture healing. The molecular response revealed that the MVFP group presented greater callus formation at the genetic level than did the LP group. Furthermore, our observations revealed that, compared with the LP group, the AMLP group expressed more osteogenesis-related genes. This finding implies that the mechanical stress induced by axial micromotion can effectively stimulate the expression of genes crucial for osteogenesis. Diverging from the AMLP group, the MVFP group successfully maintained stability at the fracture ends during the early stages. The greater expression levels of osteogenesis-related genes in the MVFP group than in the AMLP group underscore the paramount importance of early stability at the fracture ends. This result is consistent with the effects attributed to variable fixation locking screws [45]. The elevated expression levels of osteogenesis-related genes observed in this study also suggests an increased population of osteoblasts, further contributing to the understanding of the molecular mechanisms underlying the observed outcomes.

In conclusion, MVFP demonstrates the ability to achieve controlled variable fixation via a shim (providing early stable fixation and later axial micromotion fixation), making it better suited for meeting the biomechanical demands at different stages of fracture healing. Animal experiments have indicated that MVFP outperforms AMLP and LP in promoting the expression of osteogenesis-related genes. Moreover, in rabbit models, MVFP promoted faster and stronger bone healing.

It is important to acknowledge the limitations of this study. First, while the MVFP magnesium shim was very small, its potential impact on fracture healing cannot be completely ruled out. This study did not specifically investigate the influence of the plate alone on fracture healing but rather discussed the overall role of MVFP. Second, the sample size in this study was relatively small. A larger sample size would increase the credibility and statistical power of the study. Third, on the basis of the findings of this study, MVFP may be more suitable for simple extra-articular fractures of the humerus, femur, and tibia (AO type A). The suitability of MVFP for complex fractures and those occurring at other anatomical sites still requires further investigation.

## Conclusions

MVFP is a novel type of bone plate that offers variable fixation, providing both rigid initial stabilization and a transition to axial micromotion fixation. MVFP could promote faster and stronger bone healing in rabbits, potentially by accelerating the expression of BMP2 and modulating the OPG/RANKL/RANK signaling axis. This study offers valuable insights for the clinical application of variable fixation technology in bone plates and contributes to the advancement of both internal fixation technology and theory.

## Data Availability

Not applicable.

## Abbreviations

AMLP: Axial micromotion locking plate
AP: Anteroposterior
BMC: Bone mineral content
BMD: Bone mineral density
LP: Locking plate
MGV: Mean grey value
Micro-CT: Micro computed tomography
MVFP: Magnesium degradation-induced variable fixation plate
ROI: Region of interest
PVDF: Polyvinylidene difluoride
RT-PCR: Reverse transcription-polymerase chain reaction
TBST: Tris Buffered Saline with Tween 20
TGV: Total grey value
